# Comparative Effectiveness of Coronavirus Vaccine in Preventing Breakthrough Infections among Vaccinated Persons Infected with Delta and Alpha Variants

**DOI:** 10.3201/eid2802.211789

**Published:** 2022-02

**Authors:** Irina Kislaya, Eduardo Freire Rodrigues, Vítor Borges, João P. Gomes, Carlos Sousa, José P. Almeida, André Peralta-Santos, Baltazar Nunes, PT-COVID-19 Group

**Affiliations:** Instituto Nacional de Saúde Doutor Ricardo Jorge, Lisbon, Portugal (I. Kislaya, V. Borges, J.P. Gomes, B. Nunes);; Direção-Geral da Saúde, Lisbon (E.F. Rodrigues, A. Peralta-Santos);; Unilabs, Porto, Portugal (C. Sousa, J.P. Almeida)

**Keywords:** COVID-19, coronavirus disease, SARS-CoV-2, severe acute respiratory syndrome coronavirus 2, Delta variant, Alpha variant, viruses, respiratory infections, zoonoses, vaccine-preventable diseases, breakthrough infections, mRNA vaccines, Portugal

## Abstract

We developed a case–case study to compare mRNA vaccine effectiveness against Delta versus Alpha coronavirus variants. We used data on 2,097 case-patients with PCR-positive severe acute respiratory syndrome coronavirus 2 infections reported in Portugal during May–July 2021. We estimated the odds of vaccine breakthrough infection in Delta-infected versus Alpha-infected patients by using conditional logistic regression adjusted for age group and sex and matched by the week of diagnosis. We compared reverse-transcription PCR cycle threshold values by vaccination status and variant as an indirect measure of viral load. We found significantly higher odds of vaccine breakthrough infection in Delta-infected patients than in Alpha-infected patients (odds ratio 1.96 [95% CI 1.22–3.14]), suggesting lower effectiveness of the mRNA vaccines in preventing infection with the Delta variant. We estimated lower mean cycle threshold values for the Delta cases (mean difference −2.10 [95% CI −2.74 to −1.47]), suggesting higher infectiousness than the Alpha variant.

The severe acute respiratory syndrome coronavirus 2 (SARS-CoV-2) B.1.617.2 lineage, also known as Delta variant of concern (VOC), first sequenced in India in December 2020, was identified in Portugal in late April and quickly became dominant, reaching 90% of all sequenced cases in late June 2021 (epidemiologic week 26), just 2 months after it was first identified (*1*). Available evidence suggests that this VOC is associated with higher transmissibility, higher risk for hospitalization, and reduced antibody neutralization compared with other VOCs (*2*,*3*).

Vaccination is the primary pharmacologic measure to control the transmission of SARS-CoV-2 and mitigate its effect on hospitalizations and mortality rates. In Portugal, vaccination was initiated in late December 2020 for those at higher risk for severe disease or exposure and since February 2021 has been rolled out by descending age criteria. By week 26 (June 28–July 4), 36% of the population was fully vaccinated, and 56% had started or completed vaccination (Appendix Figure 1), most (75%) with mRNA vaccines (BNT162b2 [Pfizer-BioNTech, https://www.pfizer.com] or mRNA-1273 [Moderna, https://www.modernatx.com]) administrated with a 28-day dose interval (*4*).

Early reports of vaccine effectiveness indicate a high protection for mRNA vaccines against infection and disease (*5*,*6*) and a reduced viral load in the vaccinated case-patients (*7*,*8*). However, reports of vaccine effectiveness against Delta have shown decreased protection of the vaccines compared with the Alpha variant (*2*,*9*). Validating this potential reduction of vaccine effectiveness against the Delta VOC is critical to inform further public health measures, particularly as the variant becomes globally dominant. 

We aimed to provide a measure of comparative effectiveness of mRNA vaccines (BNT162b2 and mRNA-1273) against B.1.617.2 (Delta) versus B.1.1.7 (Alpha) VOCs, using a case–case study design. As a secondary objective, we compared reverse-transcription PCR (RT-PCR) cycle threshold (C_t_) values between vaccine status for Alpha and Delta variants as an indirect measure of viral load and, thus, transmissibility of the vaccine breakthrough cases for both variants.

## Methods

### Study Design

We developed an observational case–case study (*10*) comparing odds of vaccination (partial or complete) between RT-PCR–positive cases (symptomatic or asymptomatic) classified as infected with Delta versus Alpha VOCs. The study period was May 17–July 4, 2021 (epidemiologic weeks 20–26), to cover the period of VOC replacement in Portugal, from the Alpha (84.8%, week 19) to Delta dominance (96.1%, week 27) (*1*). Our analysis included persons with data on whole-genome sequencing (WGS) or spike (S) gene target failure (SGTF) who were >40 years of age and eligible for vaccination during the study period. Persons for whom data on national health registry number, age, sex, or diagnosis date were missing, and those vaccinated with Ad26.COV2-S (Johnson & Johnson/Janssen, https://www.janssen.com) or ChAdOx1 nCoV-19 (AstraZeneca, https://www.astrazeneca.com) vaccines were excluded from the study.

To indirectly infer the level of infectiousness of case-patients according to vaccination status and VOC type, we performed a secondary analysis by comparing the paired means of RT-PCR C_t_ values for nucleocapsid and open reading frame 1ab genes, by using data from a single large laboratory (Unilabs, https://unilabs.com). Lower C_t_ values reflect a reduced number of RT-PCR cycles required for amplification of SARS-CoV-2 RNA and, therefore, a higher number of virus copies within the sample. As such, studies have used C_t_ values to estimate viral load or viral shedding (*8*,*11*).

### Data Sources

#### SARS-CoV-2 Cases

RT-PCR testing for SARS-CoV-2 in Portugal is done by hospitals as well as by public and private laboratories, and is available free of charge to anyone with symptoms consistent with coronavirus disease (COVID-19) (*12*). Laboratory-confirmed cases are reported to the mandatory National Epidemiologic Surveillance Information System (Sistema Nacional de Vigilância Epidemiológica, https://www.spms.min-saude.pt/2020/07/sinave-2). For this study, each notifying laboratory selected a random subset of RT-PCR–positive nasopharyngeal samples collected during the study period to be sent to the National SARS-CoV-2 Genomic Surveillance Network (*1*,*13*) and, thus, to be included in the study. We also included samples from a private molecular biology laboratory (Unilabs) with nationwide coverage that routinely performs analysis on SGTF. We collected RT-PCR C_t_ values as an indirect measure of viral load (*11*). We removed duplicate records on the basis of national health register numbers, maintaining only the first collected sample.

#### Variant Classification

We classified SARS-CoV-2 variants by viral WGS or inferred by SGTF data. For nonsequenced samples, we considered S-positive specimens (with amplification of structural gene) as Delta and SGTF samples as Alpha by using the TaqPath COVID-19 CE IVD RT-PCR Kit (Thermo Fisher Scientific, https://www.thermofisher.com) that targets 3 genes (structural, nucleocapsid, and open reading frame 1ab), performed according to the manufacturer’s specifications, as described elsewhere (*13*).

#### Vaccination Status, Demographics, and Data Linkage

We obtained COVID-19 vaccination status through the electronic national vaccination register (https://www.sns.gov.pt/monitorizacao-do-sns/vacinas-covid-19). We classified vaccination exposure as no register of vaccine administration before diagnosis (i.e., unvaccinated); SARS-CoV-2 infection diagnosis <14 days after first dose mRNA vaccination (1 dose [<14 days]); SARS-CoV-2 infection diagnosis >14 days after first dose or <14 days after second dose (1 dose [>14 days] or 2 doses [<14 days]) (i.e., partial vaccination); and >14 days after second dose of the mRNA vaccine (2 doses [>14 days]) (i.e., complete vaccination). Information about age, sex, and date at diagnosis was routinely collected by National Epidemiologic Surveillance Information System. We performed a deterministic record linkage to join all data sources, namely on vaccination status, outcomes (VOC classification), and other covariates (e.g., age group and sex) and to remove duplicate data from the dataset.

### Statistical Analysis

We compared characteristics of delta and alpha SARS-CoV-2 case-patients by using the χ^2^ test. We considered Delta-infected case-patients as case-patients of interest and Alpha-infected case-patients as the reference group. We used conditional logistic regression matched by the week of diagnosis and adjusted for age group and sex to estimate confounder-adjusted odds of having been infected by SARS-CoV-2 and vaccinated among Delta case-patients compared with Alpha case-patients. These covariates might be associated with the probability of having been vaccinated and being exposed to the virus and type of variant.

In our analysis, odds ratio (OR) = 1 indicates no difference in odds of having been infected by SARS-CoV-2 and vaccinated and, thus, a proxy of no difference between mRNA vaccine effectiveness against the Delta versus Alpha VOC. OR >1 indicates a higher odds of having been infected by SARS-CoV-2 and vaccinated, thus lower vaccine effectiveness against the Delta versus Alpha VOC, whereas OR <1 indicates a lower odds of having been infected by SARS-CoV-2 and vaccinated among Delta case-patients and a higher vaccine effectiveness against the Delta versus Alpha VOC (Appendix).

We stratified mean and SD C_t_ values for Alpha-infected and Delta-infected case-patients on the basis of vaccination status. We evaluated differences between mean C_t_ values by vaccination status and VOC by fitting a linear multiple regression model with C_t_ values as outcome, adjusting for sex, age group, and week of case diagnosis. We included an interaction term between vaccination status and VOC type in the regression model to determine whether the effect of vaccination status on C_t_ values differed between Delta and Alpha.

### Sensitivity Analysis

To assess the change of the sampling strategy for WGS from a monthly to weekly basis, which occurred on week 21, we restricted our analysis to weeks 22–26. In addition, to assess the bias of misclassification error associated with the SGTF method (particularly in the early weeks of the study period, when overall prevalence of the Delta variant was lower and SGTF sensitivity may also have been lower), we analyzed samples identified exclusively through WGS during weeks 22–26. Finally, to address whether having been infected and vaccinated was associated with lower infectiousness in any of the studied VOCs, we restricted analysis to samples with C_t_ values <25 (*8*).

### Ethics Considerations 

Genomic surveillance of SARS-CoV-2 in Portugal is regulated by Assistant Secretary of State and Health Executive Order no. 331/2021, issued on January 11, 2021. The research on genomic epidemiology of SARS-COV-2 received the clearance of the Ethics Committee of Instituto Nacional de Saúde Doutor Ricardo Jorge on March 30, 2021.

## Results

### Main Analysis

A total of 22,784 SARS-CoV-2–positive cases were reported in Portugal during May 17–July 4, 2021, among persons >40 years of age. Of 2,097 cases included in the analysis, 966 (46.1%) were variant-classified with WGS and 1,131 (53.9%) with SGTF. During the study period, 94.7% (827/873) of the S-positive sequenced samples were confirmed as Delta and 96.9% (372/384) of SGTF samples were classified as Alpha through WGS, thus indicating that the SGTF-derived VOC classification was robust.

Among Delta case-patients, we observed a higher proportion of persons >70 years of age (p<0.001) (Table 1), and a higher proportion of vaccinated persons (p<0.001) than among the Alpha case-patients. We report a statistically significant higher odds of being partially vaccinated (OR 1.70 [95% CI 1.18–2.47]) or completely vaccinated (OR 1.96 [95% CI 1.22–3.14]) among the Delta case-patients than among the Alpha case-patients, suggesting lower mRNA vaccine effectiveness for the Delta variant (Table 2). After adjustment for age group and sex, similar estimated ORs were observed for the complete vaccination scheme (OR 1.96 [95% CI 1.43–2.69]) or for partial vaccination (OR 1.81 [95% CI 1.37–2.39]).

**Table 1:** Characteristics of patients in the study sample, by severe acute respiratory syndrome coronavirus 2 variant of concern, Portugal, epidemiologic weeks 20–26 (May 17–July 4), 2021

Characteristic	Delta (B.1.617.2), no. (%)	Alpha (B.1.1.7), no. (%)	Total, no. (%)
Overall	1,366 (100)	731 (100)	2,097 (100)
Week of diagnosis			
20	53 (4)	137 (19)	190 (9)
21	64 (5)	154 (21)	218 (10)
22	112 (8)	171 (23)	283 (13)
23	192 (14)	114 (16)	306 (15)
24	249 (18)	73 (10)	322 (15)
25	350 (26)	38 (5)	388 (19)
26	346 (25)	44 (6)	390 (19)
Age group, y			
40–49	760 (56)	378 (52)	1,138 (54)
50–69	474 (35)	313 (43)	787 (38)
>70	132 (10)	40 (5)	172 (8)
Sex			
F	707 (52)	402 (55)	1,109 (53)
M	659 (48)	329 (45)	988 (47)

**Table 2:** Crude and adjusted odds ratio of being infected with severe acute respiratory syndrome coronavirus 2 and vaccinated (odds of vaccine infection breakthrough) in Delta-infected versus Alpha-infected patients, Portugal, epidemiologic weeks 20–26 (May 17–July 4), 2021*

Vaccination status	Delta, no. (%)	Alpha, no. (%)	Crude OR (95% CI)	Confounder-adjusted† OR (95% CI)
Unvaccinated	777 (57)	517 (78)	Referent	Referent
1 dose (<14 d)	229 (17)	73 (10)	1.23 (0.83 to 1.82)	1.29 (0.85 to 1.95)	
1 dose (>14 d) or 2 doses (<14 d)‡	198 (14)	49 (7)	1.70 (1.18 to 2.47)	1.81 (1.37 to 2.39)	
2 doses (>14 d)§	162 (12)	38 (5)	1.96 (1.43 to 2.69)	1.96 (1.22 to 3.14)

*OR, odds ratio. †Adjusted for sex and age group by conditional logistic regression using week of diagnosis result as matching variable. ‡Partial vaccination. §Complete vaccination.

### Secondary Analysis

We observed statistically significant higher mean C_t_ values among those with complete vaccination compared with unvaccinated case-patients for Delta (17.7 vs. 16.5 ) as well as for Alpha (21.8 vs. 18.4) (Table 3; Figure), suggesting lower viral loads in vaccinated compared with unvaccinated case-patients for both VOCs. Although the Alpha variant cases had statistically significant confounder-adjusted C_t_ values mean difference (MD) of 4.49 (95% CI 2.07–6.91) after complete vaccination, representing an increase of C_t_ values (lower infectiousness), the Delta variant cases showed only about half of that increase, with a statistically significant confounder-adjusted C_t_ value mean difference point estimate of 2.24 (95% CI 0.85–3.64) between unvaccinated and fully vaccinated breakthrough case-patients. For partial vaccination, statistically significant differences in mean C_t_ values were observed for Alpha (MD 1.87 [95% CI 0.2–3.53]) but not for Delta cases (MD −0.15 [95% CI −0.99 to 0.96]), suggesting similar viral load between unvaccinated and partially vaccinated Delta case-patients.

**Table 3:** C_t_ values based on mean reverse-transcription PCR C_t_ values for nucleocapsid and open reading frame 1ab genes, stratified by vaccination status and severe acute respiratory syndrome coronavirus 2 variant of concern, and confounder-adjusted mean differences, Portugal, epidemiologic weeks 20–26 (May 17–July 4), 2021

Vaccination status	Delta (B.1.617.2, mean (SD)	Alpha (B.1.1.7), mean (SD)	Mean difference,* Delta vs. Alpha (95% CI)
Overall	16.4 (5.0)	18.7 (5.3)	−2.10 (−2.74 to −1.47)
Unvaccinated	16.5 (4.9)	18.4 (5.2)	−1.66 (−2.37 to −0.95)
1 dose (<14 d)	15.7 (4.9)	19.2 (5.6)	−1.42 (−3.07 to 0.23)
1 dose (>14 d) or 2 doses (<14 d)†	16.1 (5.0)	20.0 (5.6)	−1.88 (−3.77 to −0.003)
2 doses (>14 d)‡	17.7 (5.7)	21.8 (5.7)	−2.24 (−4.8 to 0.32)
Mean difference, partial vaccinated vs. unvaccinated*	−0.15 (−0.99 to 0.96)	1.87 (0.2 to 3.53)	
Mean difference, complete vaccinated vs. unvaccinated*	2.24 (0.85 to 3.64)	4.49 (2.07 to 6.91)	

*Mean difference and respective 95% CIs estimated by linear regression model adjusted to sex, age group, and week of diagnosis. †Partial vaccination. ‡Complete vaccination.

**Figure:**
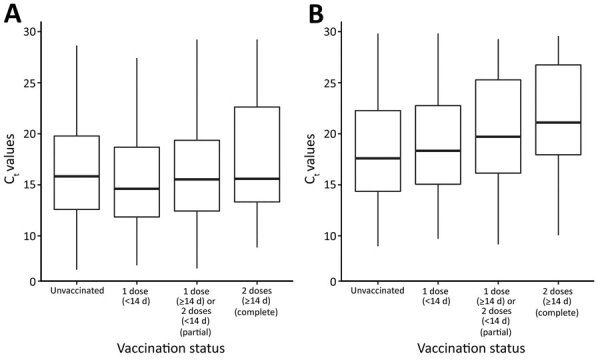
Distribution of C_t_ paired mean (reverse-transcription PCR C_t_ values for nucleocapsid and open reading frame 1ab genes) by coronavirus variant and vaccination status, Portugal, epidemiologic weeks 20–26 (May 17–July 4), 2021. A) Delta variant; B) Alpha variant. Box top and bottom indicate first and the third quartiles of C_t_ distribution, horizontal line inside box indicates median, and whiskers indicate minimum and maximum values. C_t_, cycle threshold.

The confounder-adjusted paired-mean difference in C_t_ values between Delta and Alpha was statistically significant for unvaccinated persons (MD –1.66 [95% CI –2.37 to –0.95]). For partially (MD –1.88 [95% CI –3.77 to 0.003]) and completely vaccinated persons (MD –2.24 [95% CI –4.8 to 0.32]) we did not observed statistically significant mean differences in C_t_ values between delta and alpha.

### Sensitivity Analysis

In a sensitivity analysis (Table 4), restricted to weeks 22–26, confounder-adjusted OR estimates for having been infected and completely vaccinated (OR 1.78 [95% CI 1.01–3.13]) and partially vaccinated (OR 1.91 [95% CI 1.22–3.00]) remained similar and statistically significant. Restricting the analysis to the cases identified through WGS, we observed a drop in the adjusted OR point estimate of complete vaccination (OR 1.48 [95% CI 0.75–2.93]) with a loss of statistical significance, but for partial vaccination, estimates yielded increased significant results (OR 2.5 [95% CI 1.23–5.08]). Restricting to cases with C_t_ values <25 (n = 1,363), we observed an increase of the OR of infection and vaccination against Delta versus Alpha variants both for partial (OR 2.47 [95% CI 1.48–4.12]) and complete vaccination (OR 2.42 [95% CI 1.06–5.51).

**Table 4:** Sensitivity analysis crude and adjusted odds ratio of being infected and vaccinated (odds of vaccine infection breakthrough) in Delta versus Alpha SARS-CoV-2 cases, Portugal, weeks 20–26 (May 17–July 4), 2021*

Vaccination status	Delta (B.1.617.2), no. (%)	Alpha (B.1.1.7), no. (%)	Crude OR (95% CI)	Confounder-adjusted† OR (95% CI )
Restricted to epidemiologic weeks 22–26, n = 1,689				
Unvaccinated	682 (55)	328 (75)	Referent	Referent
1 dose (<14 d)	224 (18)	54 (12)	1.34 (0.94 to 1.91)	1.38 (0.96 to 1.98)
1 dose (>14 d) or 2 doses (<14 d)‡	190 (15)	32 (7)	1.87 (1.22 to 2.86)	1.91 (1.22 to 3.00)
2 doses (>14 d)§	153 (12)	26 (6)	1.99 (1.25 to 3.16)	1.78 (1.01 to 3.13)
Restricted to WGS-classified VOC patients, n = 931				
Unvaccinated	406 (59)	189 (77)	Referent	Referent
1 dose (<14 d)	104 (15)	29 (12)	1.21 (0.75 to 1.95)	1.20 (0.73 to 1.96)
1 dose (>14 d) or 2 doses (<14 d)‡	84 (12)	11 (4)	2.63 (1.34 to 5.20)	2.50 (1.23 to 5.08)
2 doses (>14 d)§	90 (13)	18 (7)	1.91 (1.09 to 3.34)	1.48 (0.75 to 2.93)
Restricted to patients with C_t_ values <25, n = 1,363				
Unvaccinated	492 (57)	412 (82)	Referent	Referent
1 dose (<14 d)	161 (19)	48 (10)	1.20 (0.79 to 1.81)	1.30 (0.85 to 1.98)
1 dose (>14 d) or 2 doses (<14 d)‡	142 (17)	30 (6)	2.14 (1.32 to 3.46)	2.47 (1.48 to 4.12)
2 doses (>14 d)§	64 (7)	14 (3)	1.92 (0.97 to 3.82)	2.42 (1.06 to 5.51)

*C_t_, cycle threshold; OR, odds ratio; WGS, whole-genome sequencing; VOC, variant of concern. †Adjusted for sex and age group by conditional logistic regression using week of diagnosis result as matching variable. ‡Partial vaccination. §Complete vaccination.

## Discussion 

We observed statistically significant higher odds of having been infected and vaccinated (vaccine infection breakthrough) among Delta-infected versus Alpha-infected case-patients, suggesting a lower mRNA vaccines effectiveness for SARS-CoV-2 infection with the Delta VOC. The findings were consistent for both complete and partial vaccination. Delta breakthrough case-patients have a higher viral load (lower C_t_ values) compared with Alpha breakthrough case-patients.

The OR estimates for complete vaccination (vaccine infection breakthrough) (1.96) are in line with findings of test-negative design studies on vaccine effectiveness from Scotland and England (*2*,*9*) based only on SGTF or mixed SGTF and WGS methodology for variant identification. Those studies reported a 5.9 percentage point reduction of BNT162b2 vaccine effectiveness against the Delta VOC compared with 13.0 percentage points for the alpha VOC for complete vaccination (*2*,*9*), with nonoverlapping CIs for vaccine effectiveness estimates.

For partial vaccination, our results indicated statistically significant lower mRNA vaccine effectiveness against the Delta VOC (OR = 1.8), supporting the need to promptly complete vaccination schedules to account for swiftly reduced effectiveness against this variant. This result is not in line with previous research conducted in England (*9*). Several factors may explain the differences between that study (*9*) and our work: the target population is different (persons >16 years of age vs. >40 years of age); differences exist in the Alpha and Delta relative frequencies during the study period; differences exist in vaccination calendar and time between doses administration, with England having a higher proportion of persons exposed to 2 doses because of an earlier campaign roll out and Portugal having shorter time of exposure to a single dose; and a sample selection methodology for WGS.

On our secondary analysis, we observed lower C_t_ values (indicative of higher viral loads) among Delta compared with Alpha case-patients (MD −2.10 [95% CI −2.74 to −1.47]). Furthermore, although complete vaccination increases C_t_ values (thus reducing estimated viral loads) in Alpha case-patients by 4.49 (95% CI 2.07–6.91), for the Delta variant, complete vaccination had a much lower increase of C_t_ values (2.24 [95% CI 0.85–3.64]), only half of the difference observed for Alpha case-patients. These findings are consistent with vaccine infection breakthrough cases; Delta variant case-patients have higher infectiousness than alpha variant case-patients. Our findings were similar to results of the studies performed in Israel, when Alpha variant was the dominant variant, that also found a 5.09 (95% CI 2.8–7.4) increase of nucleocapsid gene C_t_ values (*8*) between completely vaccinated and unvaccinated cases and 1.51 for those partially vaccinated with BNT162b2 (*7*). Those findings are consistent with our results for the Alpha variant.

Our results were robust to variations in the sampling strategy, including changing the weeks of diagnosis included. Restricting analysis to samples with higher viral loads (selected on the basis of low C_t_ values [C_t_ <25]), we observed an even higher OR of vaccine infection breakthrough between Delta and Alpha variant cases, which supports the relevance of the Delta relative vaccine effectiveness reduction effect in the transmission of SARS-CoV-2, both for partial (OR 2.47 [95% CI 1.48–4.12]) and complete vaccination (OR 2.42 [95% CI 1.06–5.51]). The restriction to WGS samples only, however, resulted in a loss in a sample size and lack of statistical power to detect differences for complete vaccination.

This study has several limitations; in particular, possible misclassification and selection and confounding bias should be acknowledged. Although we observed a high positive predictive value (94.7%) of non-SGTF data to identify Delta cases within the study sample, a misclassification error may have led to underestimating the reported effect. However, SGTF methodology has previously shown good classification accuracy to identify B.1.1.7 in Portugal (*13*) and has more recently been shown to distinguish between Alpha and Delta VOCs in Scotland and England (*2*,*9*) and may be highly useful when a large-scale testing strategy is in place and electronic vaccination registers are used. In addition, information bias caused by misclassification of vaccination status can arise from delays in registering vaccination status and diagnosis. However, data extraction was performed 3 weeks after the end of the study period to minimize bias (e.g., vaccination centers are instructed to register vaccinations up to 24 hours after administration). Our sampling strategy resulted in some selection bias. Overall, 22,784 cases were identified in Portugal during the study period among those >40 years of age, and their age distribution was different from the study sample (Appendix Figure 4), skewed toward younger ages in the study sample, possibly because our sample was collected mainly through ambulatory laboratories, whereas older persons (>80 years of age) are expected to be more frequently diagnosed by hospital laboratories. This result could bias our estimates if the reduction of vaccine effectiveness between Delta and Alpha variant is age-dependent. For example, if the vaccine effectiveness reduction is higher among older persons, our results could be underestimated. Moreover, Delta cases occurred more frequently among older participants, and older participants were the first to be vaccinated in Portugal (*4*) and, thus, had a longer exposure time after their second dose. With time, a waning of the vaccine effect can occur, possibly contributing to the observed differences among persons who were completely vaccinated.

After adjusting for confounding, we did not observe a substantial change in OR estimates. Although we cannot exclude residual confounding bias, given that other factors not accounted for might be associated with the probability of exposure to the virus and of being vaccinated (e.g., health and social services worker status, ethnicity, and education). However, we found that other studies (*9*) that adjusted for these potential confounding factors not accounted in our study did not observe a substantial difference between crude and confounder-adjusted estimates. Hence, if any residual confounding occurred, it could have a small effect on the effect estimates.

The results must always be interpreted in context because they do not provide evidence to question the benefits of the mRNA vaccines to individual health, such as reducing symptoms, disease severity, or the impact on health services capacity. We reported odds of vaccine breakthrough between Delta and Alpha VOCs, which can be interpreted as a measure of the relative vaccine effectiveness. Although a case–case study design does not provide a direct measure of effectiveness against a specific VOC, it may be useful for rapidly detect changes in vaccine effectiveness in the context of novel VOC emergence, providing substantial evidence for further public health measures to control the transmission of SARS-CoV-2.

Overall, we found significantly higher odds of vaccination in Delta case-patients than in Alpha case-patients, suggesting possible lower effectiveness of the mRNA vaccines in preventing infection with the Delta VOC. Case–case design has proven to be helpful to compare vaccine effectiveness for SARS-CoV-2 VOCs because of its quick implementation and valuable insights in the context of frequent and swift VOC emergence. These findings can help decision-makers as they consider applying or lifting of control measures and adjusting vaccine roll-out depending on the predominance of the Delta variant and levels of partial and complete mRNA vaccination coverage.

Appendix Additional information about comparative effectiveness of coronavirus vaccine in preventing breakthrough infections among vaccinated persons infected with delta and alpha variants.
