# Supplementary material for: Comparative Effectiveness of Coronavirus Vaccine in Preventing Breakthrough Infections among Vaccinated Persons Infected with Delta and Alpha Variants
Source: Emerg Infect Dis. 2022 Feb;28(2):331–7. doi: 10.3201/eid2802.211789 (PMC8798697; doi:10.3201/eid2802.211789)
Supplement: Appendix — Additional information about comparative effectiveness of coronavirus vaccine in preventing breakthrough infections among vaccinated persons infected with delta and alpha variants. [file 21-1789-Techapp-s1.pdf]

Article DOI: https://doi.org/10.3201/eid2802.211789

Comparative Effectiveness of Coronavirus Vaccine in Preventing Breakthrough Infections among Vaccinated Persons Infected with Delta and Alpha Variants

Appendix

Case–Case Study Design Description

The most common approach of vaccine effectiveness (VE) studies is test-negative case-control (TND) design, where VE is estimated comparing odds of vaccination between positive (cases) and negative (controls) (VE = (1 – OR) × 100%).

Variant-specific vaccine effectiveness estimates in TND studies are obtained restricting comparisons to cases positive to specific variant versus negative controls (Appendix Table) resulting in vaccine effectiveness estimates:

$VE_{Delta} = (1 - OR_{Delta}) \times 100\%$ and, $VE_{Alpha} = \left(1 - OR_{Alpha}\right) \times 100\%$,

where OR_{Delta} and OR_{Alpha} are obtained as follows:

$$OR_{Delta} = \frac{a \times d}{c \times b} \; OR_{Alpha} = \frac{k \times d}{m \times b}$$

Only positive cases are considered in case–case design, and odds of vaccination are compared directly between two variants. Considering Delta cases as cases of interest and Alpha cases as a reference group, the case–case OR is estimated as:

$$OR_{case-case} = \frac{a \times m}{c \times k}$$

that is mathematically equivalent to the ratio of two variant-specific OR obtained from TND:

$$\frac{OR_{Delta}}{OR_{Alpha}} = \frac{a \times d}{c \times b} \div \frac{k \times d}{m \times b} = \frac{a \times d \times m \times b}{c \times b \times k \times d} = \frac{a \times m}{c \times k} = OR_{case-case}$$

The case–case approach allows to formally test whether the difference between variant-specific odds ratios is statistically significant and infer variant-specific vaccine effectiveness (Appendix Figure 5).

Appendix Table. Classification of cases by vaccination and positivity status

Vaccination status	Delta+	Alpha+	Negative controls
Vaccinated	a	k	b
Unvaccinated	c	m	d

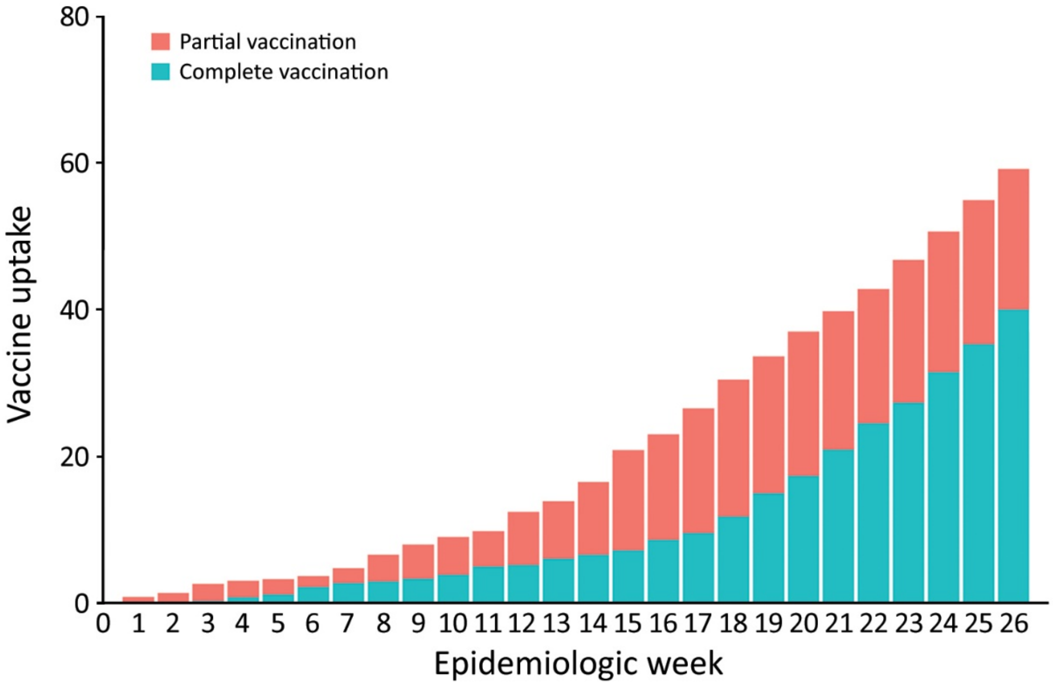

Appendix Figure 1. Vaccine uptake, from week 1 to 26 of 2021 in mainland Portugal.

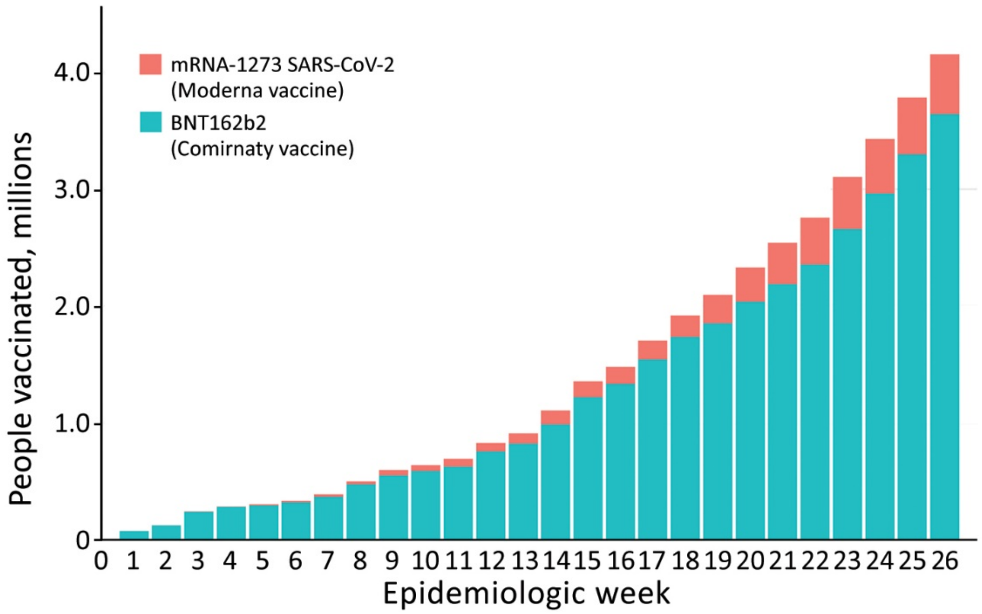

Appendix Figure 2. Number of people vaccinated with mRNA vaccine by brand, from week 1 to 26 of 2021 in mainland Portugal.

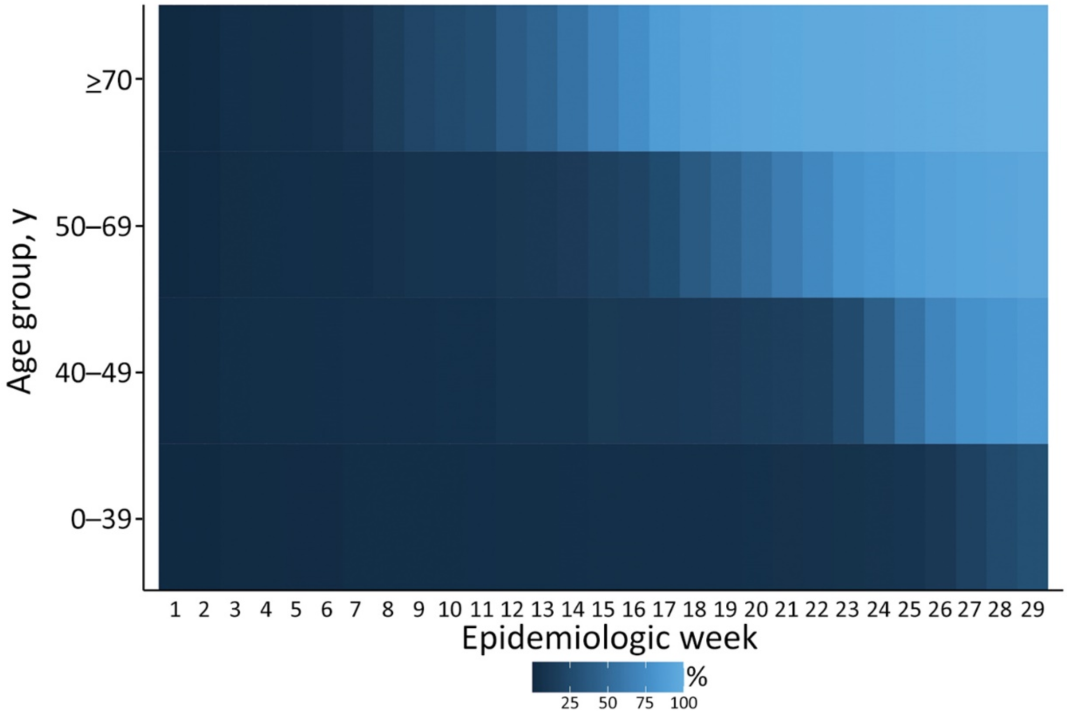

Appendix Figure 3. mRNA vaccine coverage per age group and week, from week 1 to 26 of 2021 in mainland Portugal.

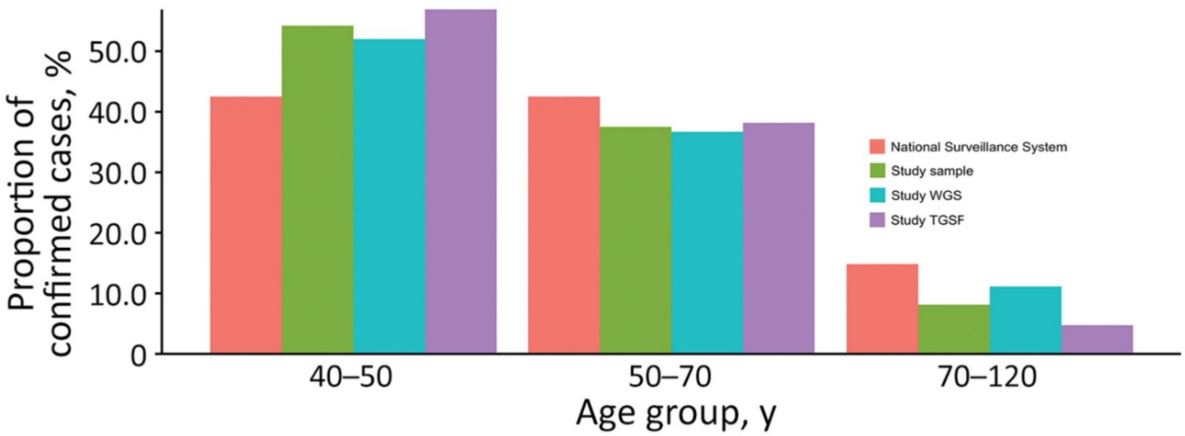

Appendix Figure 4. Relative frequency of age groups of reported confirmed cases over 40 years to the national surveillance system and study sample, from week 20 to 26 of 2021 in mainland Portugal.

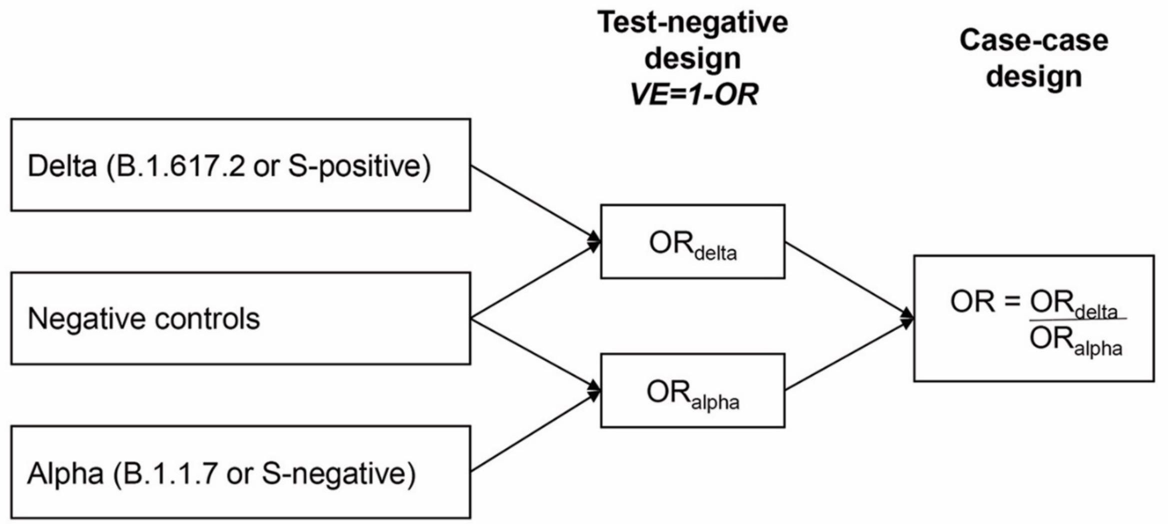

Appendix Figure 5. Comparison diagram between test-negative design and case–case design.
